# Changing Sodium Knowledge, Attitudes and Intended Behaviours Using Web-Based Dietary Assessment Tools: A Proof-Of-Concept Study

**DOI:** 10.3390/nu11092186

**Published:** 2019-09-11

**Authors:** Katherine Jefferson, Zhila Semnani-Azad, Christina Wong, Mary R. L’Abbé, JoAnne Arcand

**Affiliations:** 1 Faculty of Health Sciences, Ontario Tech University, 2000 Simcoe St. North, Science Building, Rm 3016, Oshawa, ON L1G 0C5, Canada; katherine.jefferson@uoit.ca; 2 Department of Nutritional Sciences, University of Toronto, Medical Sciences Building, 1 King’s College Circle, Toronto, ON M5S 1A8, Canada; z.semnani.azad@mail.utoronto.ca (Z.S.-A.); laikuen@gmail.com (C.W.); mary.labbe@utoronto.ca (M.R.L.)

**Keywords:** sodium, dietary assessment, knowledge, attitudes and behaviours, sodium policy

## Abstract

Despite public health efforts to reduce dietary sodium, sodium intakes in most countries remains high. The purpose of this study was to determine if using novel web-based tools that provide tailored feedback, the Sodium Calculator and Sodium Calculator Plus, improves users’ sodium-related knowledge, attitudes, and intended behaviours (KAB). In this single arm pre- and post-test study, 199 healthy adults aged 18–34 years completed a validated questionnaire to assess changes to sodium-related KAB before and after using the calculators. After using the calculators, the proportion of participants who accurately identified the sodium adequate intake and chronic disease risk reduction level increased (19% to 74% and 23% to 74%, respectively, both *p* = 0.021). The proportion accurately self-assessing their sodium intake as ‘high’ also increased (41% to 66%, *p* = 0.021). Several intended behavioural changes were reported, i.e., buying foods with sodium-reduced labels, using the Nutrition Facts table, using spices and herbs instead of salt, and limiting eating out. Evidence-based eHealth tools that assess and provide personalized feedback on sodium intake have the potential to aid in facilitating sodium reduction in individuals. This study is an important first step in evaluating and optimizing the implementation of eHealth tools to help reduce Canadians’ sodium intakes.

## 1. Introduction

Hypertension is a major risk factor for cardiovascular disease, stroke and kidney disease, and is the leading preventable risk factor for death worldwide [1]. Excess dietary sodium is responsible for approximately 30% of cases of hypertension [2]. In developed countries, sodium intakes far exceed recommendations. On average, Canadians consume 2760 mg of sodium per day, an intake far exceeding the adequate intake (AI) (1500 mg) and chronic disease risk reduction intake (CDRR) (2300 mg) levels [3]. Notably, 90% of males between 14–30 years of age consume sodium at levels beyond these recommendations [4]. To address the disease burden associated with excess sodium consumption, the World Health Organization set a global target of reducing dietary salt intake by 30% by 2025 [5].

In response, many countries have implemented population-wide sodium reduction strategies [6]. However, from a socio-ecological perspective, the implementation of recommendations requires policy action at both the population and individual level [7]. For example, Canada’s Sodium Reduction Strategy includes several recommendations aimed at sodium reduction in the food supply, as most dietary sodium is derived from packaged foods [8]. However, two recent evaluations showed minimal progress has been made by the food industry in effectively reducing sodium levels [9,10]. This illustrates the need for concurrent action by individuals in reducing dietary sodium. Indeed, for public policies to be effective, individuals must have an appreciation for how a particular issue affects them [11,12]. This is supported by the theory of planned behaviour, which predicts that attitudes or beliefs about the outcome of the behaviour influences actions or behaviours [13]. This is especially relevant because there are many misconceptions and lack of knowledge about dietary sources of sodium that may impede effective action in sodium reduction. While population health approaches are critical, individualized interventions and counseling are cost effective (<$50,000 USD/DALY) in chronic disease prevention and management [14].

In response to these challenges, two dietary sodium eHealth tools were created: the Sodium Calculator (SC) and Sodium Calculator Plus (SCP) [15]. These tools were developed to provide a user with instant, individualized feedback on estimated daily sodium intake, a comparison of user’s sodium intake with the recommendations, and a report on the main sources of dietary sodium. These tools were developed to address inherent limitations with existing dietary assessment tools: 24 h urine collections, food frequency questionnaires, multiple-day food records and 24 h food recalls, as these can be time-consuming, burdensome, not clinically feasible and may not provide timely feedback [16,17]. The SC and SCP were developed based on the premise that consumers would benefit from understanding their personal sodium intake in order to take personal action on sodium reduction [12]. They ask questions on type and frequency of consumption of high sodium foods, with the SCP collecting more detailed information on portion sizes and use of reduced/low sodium options. The provision of tailored feedback is known to increase awareness of personal behaviour patterns, personal behaviours compared to recommendations, and assist with setting and monitoring progress towards behaviour change goals [18,19,20]. Individualized feedback also makes communicated information more likely to be read, remembered, discussed, and viewed as interesting [21], and thus, it increases the likelihood that an individual will show an interest in adopting behaviors to reduce their dietary sodium [22].

Although these promising publicly-available tools exist, the impact of the Sodium Calculators on individuals’ knowledge, attitudes and intended behaviour (KAB) remains unknown. Therefore, the purpose of this study was to determine whether dietary sodium-related KAB improves after using the Sodium Calculators in a cohort of healthy, young adults. This objective is situated within the ORBIT model of behavioural interventions as a first stage proof-of-concept pilot study examining the efficacy of the Sodium Calculators to optimize the intervention for more rigorous evaluation as part of a future randomized controlled trial [23]. It is hypothesized that the use of the Sodium Calculators will improve nutrition knowledge, attitudes and intended behaviours due to the tailored individualized feedback provided by the tools.

## 2. Materials and Methods

### 2.1. Subjects and Study Design

In this single arm pre- and post-test study, a sample of healthy, young adults, aged 18–35 years were recruited using email, class announcements in undergraduate classes and social media from January 2014 to April 2014. Subjects were fluent in English and had no clinical diagnoses (i.e., hypertension, heart or renal failure) that required a low sodium diet. Subjects also did not consume more than two meals/day from food service establishments. Eligible participants who provided informed consent were assessed at a research laboratory where they independently completed a KAB questionnaire, which was administered before and after completing the online Sodium Calculators. This was a sub-study of a larger trial to validate the calculators against other dietary sodium assessment tools, and therefore, the SC and SCP were completed consecutively in random order. On average, participants took 10–15 min to complete the calculator assessments. Estimated sodium results from only the second completed calculator was shown to the participants. The protocol was approved by the Research Ethics Board at the University of Toronto (REB Protocol #29339).

### 2.2. Study Materials

Sodium Calculators. The SC and SCP were developed to fill a gap in user-friendly tools that provide timely assessment and feedback of sodium intake (www.projectbiglife.ca), with the design and development details previously published [11]. Briefly, the SC contains 23 questions about the frequency of sodium-containing foods that takes under 5 min to complete. The SCP includes the same 23 questions but asks additional questions to capture other sources of dietary sodium. The SCP contains 66 questions in total, including questions on the use of low-sodium versions of packaged foods. While the SC portion sizes are based on average portions consumed by 13 different age and sex groups, the SCP asks about typical portion sizes consumed. Both were developed considering national data on the sources of sodium in the Canadian diet from the Canadian Community Health Survey [24], and the sodium content of processed and restaurant foods and portion sizes according to a University of Toronto database containing the nutrient content of foods in the Canadian food supply [10,25,26]. Both calculators generate personalized detailed user reports that include the estimated amount of sodium consumed (mg/day) with a comparison of the user’s estimated intake to the recommended AI and CDRR for their age and gender. The reports also include a ranking and the relative percent contribution of sodium from foods consumed [11]. A more detailed description of the SC and SCP can be found elsewhere [11].

KAB Questionnaire. The KAB questionnaire contained 10 questions to assess user knowledge (two questions), attitudes and interest (four questions) in sodium reduction, and intended behaviours (four questions; two of which contained seven and six sub-questions on sodium reduction strategies, respectively), and was administered as a paper hard copy. These questions were selected from a validated survey that had previously been administered to a nationally representative sample of the Canadian population [15]. The questionnaire was a mix of nominal (multiple choice) and Likert scale questions (e.g., 1 = very little, 3 = moderate, 5 = a great deal). Most questions included an option of “I don’t know” or “not applicable.” Participants could only choose one answer per question.

### 2.3. Statistical Analysis

Data are presented as frequencies, percentages and standard errors for categorical variables and means and standard errors for continuous variables. Rao–Scott Chi-Square tests assessed the differences in KAB before and after the intervention for categorical variables. Likert scale responses that included ‘don’t know’ or ‘not applicable’ were excluded from the analysis. Multiple comparisons were accounted for using the Holm–Bonferroni correction. A *p*-value of <0.05 was considered statistically significant. SAS version 9.3 (SAS Institute, Cary, NC, USA) was used for the statistical analyses.

## 3. Results

Participants (*n* = 199) were 21 ± 3 years and 49% male. On average, participants reported eating out three times a week. Six percent lived in a university campus residence. Estimated mean sodium intake was 3656 ± 267 mg/day by the SCP, with 96% and 81% of subjects consuming excess sodium intake as compared to the adequate intake and tolerable upper level, respectively.

### 3.1. Knowledge

After using the calculators, sodium knowledge improved. Knowledge of the sodium AI recommendation increased from 19% to 74% (*p* = 0.021) and knowledge of the CDRR level increased from 23% to 74% (*p* = 0.021) (Table 1). Additionally, use of the calculators reduced the number of participants who stated they did not know the AI (33% to 9%, *p* = 0.021) and CDRR level (37% to 8%, *p* = 0.021) (data not shown).

### 3.2. Attitudes and Interest in Lowering Sodium

At baseline, the majority of participants believed that dietary sodium affects their health either ‘a lot’ or ‘a great deal’, but this was not significantly higher after the intervention (75% to 82%, *p* = 0.100) (Table 1). Importantly, the number of participants who accurately reported their sodium intake as high or low also increased from 53% to 76% (*p* = 0.021), and there was a significant increase in the number who thought they consumed more sodium than the average Canadian (28% to 52%, *p* = 0.021) (Table 1). After using the calculators, the number of participants who believed they did not need to limit sodium intake decreased from 37% to 23% (*p* = 0.045). Additionally, there was an increase in interest towards reducing sodium intake from 33% to 48% (*p* = 0.045) (Table 1). Although these were not statistically significant findings, they show a practical and potential clinically meaningful trend towards improvement in belief and interest in sodium reduction.

### 3.3. Intended Behaviours Regarding Sodium Reduction

After using the calculators, participants reported that they intended to change several behaviours to lower their sodium intake (Table 2), indicating that they would ‘mostly’ or ‘always’ buy foods with sodium reduced labels (36% to 72%, *p* = 0.021), use the Nutrition Facts table (40% to 75%, *p* = 0.021), use spices, herbs, seasoning instead of salt 52% to 81%, *p* = 0.02), request sauces on the side at restaurants (21% to 50%, *p* = 0.021), limit or avoid eating out (35% to 60%, *p* = 0.021), and rinse canned vegetables before use (46% to 70%, *p* = 0.021). Avoiding the use of salt during cooking and at the table were ranked lowest among all intended behaviour changes but was still greater after use of the calculators (35% to 47%, *p* = 0.021 and 60% to 73%, *p* = 0.100, respectively).

## 4. Discussion

This study showed that simple eHealth tools that assess and provide feedback on dietary sodium intake may improve users’ KAB related to dietary sodium. We hypothesize that this observation was attributable to the immediate, personalized feedback and awareness that users experience upon completion of the SC and SCP. The results of this proof-of-concept study is an important step in the evaluation and optimization of novel eHealth interventions, as contextualized as part of the ORBIT model for the design and evaluation of behavioural interventions [23]. This data demonstrate that the Sodium Calculators merit continued testing as part of a larger randomized controlled trial due to the clinically relevant improvements in KAB among individuals that used the calculators [23].

Such tools are highly relevant to support the implementation of sodium reduction strategies, which is a public health priority globally. In Canada, this type of health behaviour research is warranted because of documented misconceptions about dietary sodium. While most Canadians understand the negative health impacts of excess sodium, there remains a disconnect between this knowledge and personal action. For example, one study showed that 89% of Canadians accurately believe that the overall population consumes too much sodium, but 59% felt that their personal sodium intake was within the recommendations, contrary to national sodium intake surveillance data [27]. In a nationally representative survey conducted by our group, many Canadians who were taking action to lower sodium in their diet felt that they consumed low amounts of sodium because they do not add salt to their food at the table or during cooking, a common misconception [15]. Addressing and ameliorating misconceptions about dietary sodium is the first step to enabling individuals in taking effective personal action in sodium reduction.

Previous research has shown that the more relevant a health message is to an individual, the more intently and positively the information will be perceived [20,28,29,30]. The personalized feedback provided from the calculators likely explains the increase in the number of participants who accurately recalled the sodium recommendations and indicated intended behaviour changes to reduce dietary sodium. This study observed a 43% increase in the number of participants who accurately reported their sodium intake as high or low after using the calculators. Tailored messages are significantly more effective at facilitating behavioural change compared to non-tailored messages [31], and have been successfully used in interventions targeting other health behaviours such as smoking, alcohol intake, fruit and vegetable intake and physical activity [31]. Likewise, providing feedback via a ranking of the main contributors of sodium in the user’s diet allows for users to plan personal strategies for action by targeting specific foods to limit, which in theory will have a meaningful impact on sodium intake.

When examining the impact of the SC and SCP in changing attitudes related to sodium intake, it is important to consider the age of the users. Younger adults tend to underestimate their cardiovascular disease risk and perceive heart disease to be a lower risk than other chronic diseases such as cancer [4,32]. This warrants an examination among an older age cohort, especially those with present risk factors for cardiovascular disease. Interestingly, however, the young adults in our study reported that they intended to implement several behaviour changes—many of which would be effective at helping them reduce their sodium. It is noteworthy that “not adding salt” to food using a salt shaker was among the lowest ranked intended behaviour changes, suggesting that the participants either learned that sodium added through salt is not a principle source of sodium intake (a common misconception) or were already not engaging in this behaviour.

While the SC and SCP can be used by individuals, they may also be used as a decision-support tool by health care professionals. For individuals, interactions with the health care team is a critical point of intervention for targeting health behaviours like dietary sodium; however, physician implementation of dietary recommendations and advice is limited due to several barriers (knowledge, time, self-efficacy, etc.) [33,34]. Nutrition eHealth innovations are low cost, feasible and highly regarded by physicians to be a facilitator in mitigating challenges associated with providing dietary advice [35]; therefore, it would be expected that they would optimize communication, shared decision making and overall quality of care in patient–provider interactions. In this emerging area of nutrition science, whether eHealth tools are effective at improving the quality of nutrition care provided by healthcare providers, and in facilitating patient behaviour change, is unknown. Thus, the SC and SCP may have particular relevance in clinical settings that manage patients with hypertension, cardiovascular disease and chronic kidney disease, which should be the subject of future research.

There are limitations to this study that should be noted. Because this study was a sub-study of a larger clinical study, assessing which calculator had a greater impact on KAB could not be determined. However, it should be noted that the SC and SCP are derivatives of each other and there are several overlapping questions. This study did not include a control group. However, this was a single-arm pre/post, proof-of-concept study, as defined in Phase II of the ORBIT model [23]. These types of studies generate data using quasi experimental or pre/post designs (no control group) that aim to examine if behavioural interventions produce a clinically meaningful change in a behavioural risk factor. These studies are essential to elucidate if an intervention merits further investigation as part of a more resource-intensive larger behavioural intervention randomized controlled trial. This study also did not measure changes to dietary sodium intake or other clinical outcomes. We chose to measure sodium-related knowledge, attitudes and intended behaviour change (KAB). A change in KAB does not necessarily guarantee positive behaviour change; however, it is necessary for effective behaviour change to occur and therefore is clinically relevant. This choice of outcome is further supported by the theory of planned behaviour, which predicts that attitudes or beliefs about the outcome of the behaviour influences actions or behaviours [13].

## 5. Conclusions

Improving KAB related to dietary sodium is a key component of population-wide sodium reduction strategies [11,12]. This proof-of-concept study in a group of university students found that simple eHealth tools, the SC and SCP, increase knowledge of sodium recommendations, improve awareness of personal sodium intake and increase behavioural intentions in reducing sodium intake. This is likely due to the immediate, tailored feedback provided. Whether or not the SC and SCP promote change in individual dietary habits is currently being investigated. We have shown that the SC and SCP are feasible, low cost and potentially clinically relevant interventions. The data point to the appropriateness and need to move forward with a fully powered RCT to examine the long-term impact of these interventions on clinical outcomes, including objective (laboratory) measures of sodium intake.

## Figures and Tables

**Table 1:** Participant sodium-related knowledge and attitudes.

(*n* = 199)	Before Intervention % (SE)	After Intervention % (SE)	*p* Value
Correctly reported the Tolerable Upper Level	23.1 (3.0)	74.4 (3.1)	0.021
Correctly reported the Adequate Intake level	19.1 (2.8)	74.4 (3.1)	0.021
Belief about the degree to which sodium affects health			
Very little	3.7 (1.4)	2.4 (1.2)	0.100
Little	4.7 (1.5)	4.1 (1.5)	
Moderate	16.3 (2.7)	11.8 (2.5)	
A lot	46.8 (3.6)	39.1 (3.8)	
A great deal	28.4 (3.3)	42.6 (3.8)	
Belief about how one’s intake compares to the Canadian population			
Much lower	7.5 (1.9)	6.5 (1.8)	0.021
Somewhat lower	35.2 (3.4)	20.6 (2.9)	
About the same	25.1 (3.1)	19.6 (2.8)	
Somewhat higher	22.6 (3.0)	28.1 (3.2)	
Much higher	5.5 (1.6)	24.1 (3.0)	
Don’t know	4.0 (1.4)	1.0 (0.7)	
Belief about the amount of sodium consumed			0.021
Far too much	41.4 (3.5)	65.8 (3.4)	
The right amount	45.5 (3.5)	30.2 (3.3)	
Far too little	8.1 (1.9)	3.0 (1.2)	
Don’t know	5.1 (1.6)	1.0 (0.7)	
Action towards reducing dietary sodium			
Don’t need to limit	14.6 (2.5)	9.6 (2.1)	0.045
Not interested in limiting	22.7 (3.0)	13.6 (2.4)	
Interested in limiting	32.8 (3.3)	47.5 (3.6)	
Tried to limit before	5.6 (1.6)	4.0 (1.4)	
Currently trying to limit	24.2 (3.0)	25.3 (3.1)	

*p* values were adjusted for multiple comparisons using the Holm–Bonferroni method.

**Table 2:** Engagement in intended behaviours to reduce dietary sodium before and after using the sodium calculators.

(*n* = 199)		Never % (SE)	Infrequently % (SE)	Sometimes % (SE)	Mostly % (SE)	Always % (SE)	*p* Value	N/A % (SE)
Buy foods with sodium reduced labels	Pre	26.3 (3.1)	14.6 (2.5)	19.2 (2.8)	18.7 (2.8)	17.7 (2.7)	0.021	3.5 (1.3)
	Post	8.0 (1.9)	4.0 (1.4)	14.1 (2.5)	33.7 (3.4)	38.7 (3.5)		1.5 (0.9)
Use the Nutrition Facts Table	Pre	28.8 (3.2)	14.6 (2.5)	15.2 (2.6)	19.2 (2.8)	21.2 (2.9)	0.021	1.0 (0.7)
	Post	8.1 (1.9)	6.1 (1.7)	10.2 (2.2)	22.8 (3.0)	51.8 (3.6)		1.0 (0.7)
Use spices, herbs, seasoning instead of salt	Pre	9.6 (2.1)	8.6 (2.0)	24.2 (3.0)	23.2 (3.0)	28.8 (3.2)	0.021	5.6 (1.6)
	Post	4.0 (1.4)	4.0 (1.4)	7.0 (1.8)	13.1 (2.4)	67.8 (3.3)		4.0 (1.4)
Request sauces on the side at restaurants	Pre	49.0 (3.6)	14.1 (2.5)	11.1 (2.2)	9.1 (2.0)	12.1 (2.3)	0.021	4.5 (1.5)
	Post	16.6 (2.6)	13.6 (2.4)	16.1 (2.6)	22.6 (3.0)	27.6 (3.2)		3.5 (1.3)
Limit or avoid eating out	Pre	12.6 (2.4)	22.2 (3.0)	28.3 (3.2)	22.7 (2.3)	12.1 (1.0)	0.021	2.0 (1.2)
	Post	3.0 (1.2)	10.1 (2.1)	25.3 (3.1)	25.8 (3.1)	34.3 (3.4)		1.5 (0.9)
Rinse canned vegetables before use	Pre	13.7 (2.5)	8.1 (1.9)	8.1 (1.9)	8.6 (2.0)	37.1 (3.4)	0.021	24.4 (3.1)
	Post	4.0 (1.4)	3.0 (1.2)	6.5 (1.8)	18.6 (2.8)	51.8 (3.5)		16.1 (2.6)
Taste food before adding salt	Pre	9.1 (2.1)	8.6 (2.0)	18.3 (2.8)	12.7 (2.4)	46.7 (3.6)	0.021	4.6 (1.5)
	Post	4.0 (1.4)	4.0 (1.4)	7.0 (1.8)	13.1 (2.4)	67.8 (3.3)		4.0 (1.4)
At restaurants ask for food with no added salt	Pre	85.4 (2.5)	7.1 (1.8)	2.0 (1.0)	1.0 (0.7)	1.0 (0.7)	0.021	3.5 (1.3)
	Post	35.7 (3.4)	17.1 (2.7)	21.6 (2.9)	12.1 (2.3)	11.1 (2.2)		2.5 (1.1)
Eat fewer packaged foods	Pre	7.1 (1.8)	13.1 (2.4)	19.7 (2.8)	32.3 (3.3)	24.7 (3.1)	0.021	3.0 (1.2)
	Post	3.0 (1.2)	4.0 (1.4)	13.1 (2.4)	25.1 (3.1)	49.7 (3.5)		5.0 (1.6)
Eat more vegetables and fruit	Pre	3.5 (1.3)	6.6 (1.8)	18.2 (2.7)	28.3 (3.2)	43.4 (3.5)	0.021	0 (0)
	Post	1.0 (0.7)	3.0 (1.2)	6.6 (1.8)	18.3 (2.8)	71.1 (3.2)		0 (0)
Make meals at home	Pre	20.3 (2.9)	12.2 (2.3)	19.3 (2.8)	25.4 (3.1)	16.8 (2.7)	0.021	6.1 (1.7)
	Post	8.5 (2.0)	8.5 (2.0)	22.6 (3.0)	21.6 (2.9)	34.2 (3.4)		4.5 (1.5)
Avoid salt at table	Pre	17.8 (2.7)	8.1 (1.9)	8.6 (2.0)	11.2 (2.2)	48.7 (3.6)	0.100	5.6 (1.6)
	Post	8.6 (2.0)	4.5 (1.5)	8.1 (1.9)	12.6 (2.4)	60.6 (3.5)		5.6 (1.6)
Avoid adding salt during cooking	Pre	22.7 (3.0)	20.7 (2.9)	17.7 (2.7)	18.2 (2.7)	16.7 (2.7)	0.021	4.0 (1.4)
	Post	12.1 (2.3)	13.1 (2.4)	25.1 (3.1)	24.1 (3.0)	23.1 (3.0)		2.5 (1.1)

*p* values were adjusted for multiple comparisons using the Holm–Bonferroni method.
